# The haplotypes of various TNF related genes associated with scleritis in Chinese Han

**DOI:** 10.1186/s40246-020-00296-y

**Published:** 2020-12-07

**Authors:** Yingnan Gao, Liping Du, Fuzhen Li, Jiadong Ding, Geng Li, Qingfeng Cao, Na Li, Guannan Su, Aize Kijlstra, Peizeng Yang

**Affiliations:** 1 Department of Ophthalmology, The First Affiliated Hospital of Zhengzhou University, Henan Province Eye Hospital, Henan International Joint Research Laboratory for Ocular Immunology and Retinal Injury Repair, Jianshe East Road 1, Zhengzhou, 450052 People’s Republic of China; 2 The Academy of Medical Sciences, Zhengzhou University, Zhengzhou, People’s Republic of China; 3 The First Affiliated Hospital of Chongqing Medical University, Chongqing Key Laboratory of Ophthalmology and Chongqing Eye Institute, Youyi Road 1, Chongqing, 400016 People’s Republic of China; 4 University Eye Clinic Maastricht, Maastricht, Limburg the Netherlands

**Keywords:** Scleritis, TNF related genes, Haplotype, Gene polymorphism, A case-control study

## Abstract

**Background:**

Several studies have stated that TNF-α participates in the pathogenesis of scleritis, but also in several systemic autoimmune diseases and vasculitis, of which some are associated with scleritis. Earlier GWAS and SNP studies have confirmed that multiple SNPs of TNF related genes are associated with many immune-mediated disorders. The purpose of this study was to examine the association of TNF related gene polymorphisms with scleritis in Chinese Han. A case-control study was carried out in 556 non-infectious scleritis cases and 742 normal controls. A total of 28 single-nucleotide polymorphisms (SNPs) were genotyped by the iPLEXGold genotyping assay.

**Results:**

No significant correlations were seen between the individual SNPs in the TNF related genes and scleritis. Haplotype analysis showed a significantly decreased frequency of a TNFAIP3 TGT haplotype (order of SNPs: rs9494885, rs3799491, rs2230926) (Pc = 0.021, OR = 0.717, 95% CI = 0.563–0.913) and a significantly increased frequency of a TNFSF4 GT haplotype (order of SNPs: rs3850641, rs704840) (Pc = 0.004, OR = 1.691, 95% CI = 1.205–2.372) and TNFSF15 CCC haplotype (order of SNPs: rs6478106, rs3810936, rs7865494) (Pc = 0.012, OR = 1.662, 95% CI = 1.168–2.363) in patients with scleritis as compared with healthy volunteers.

**Conclusions:**

This study reveals that a TGT haplotype in TNFAIP3 may be a protective factor for the development of scleritis and that a GT haplotype in TNFSF4 and a CCC haplotype in TNFSF15 may be risk factors for scleritis in Chinese Han.

## Background

Scleritis is a relatively uncommon and heterogeneous ocular disorder which is clinically characterized by the insidious onset of ocular redness, pain, and tenderness [1]. According to the anatomic location, it is usually classified into anterior, posterior, or pan-scleritis [2]. Scleritis is an inflammation of the stroma of the sclera. The inflammatory process of the sclera may extend to adjacent tissues if not treated adequately, possibly causing several ocular complications and even blindness [3, 4]. However, the precise etiologic pathways or mechanisms of scleritis are not yet clear and a number of studies have indicated that an immune imbalance triggered by environmental factors may contribute to chronic inflammation of the sclera in genetically predisposed individuals [5, 6].

Several studies have stated that TNF-α participates in the pathogenesis of scleritis [7–9], but also in several systemic autoimmune diseases and vasculitis [10–12], of which some are associated with scleritis [1, 13, 14]. Earlier GWAS and SNP studies have confirmed that multiple SNPs of TNF related genes are associated with many immune-mediated disorders, including ankylosing spondylitis (AS) [15], rheumatoid arthritis (RA) [16–18], multiple sclerosis (MS) [19], inflammatory bowel disease (IBD) [20–23], psoriasis [24], systemic sclerosis (SSc) [25–27], and systemic lupus erythematosus (SLE) [28–30]. Whether TNF related gene polymorphisms are associated with scleritis has not yet been reported and was therefore the subject of the study reported here. A case-control study was made to investigate the association of genetic polymorphisms in TNF related genes with scleritis, whereby we included 28 SNPs that had previously been demonstrated to be related with autoimmune or autoinflammatory diseases.

## Results

### Clinical features of the enrolled cases and controls

The demographic and clinical characteristics of the 556 scleritis cases and 742 included volunteers are shown in Table 1.

**Table 1:** Clinical manifestations of scleritis patients and controls who participated in this research

Clinical features	North of the Yangtze River		South of the Yangtze River		Total	
	*N*	%	*N*	%	*N*	%
Patients with scleritis	291		265		556	
Ethnicity	Han Chinese		Han Chinese		Han Chinese	
Mean age ± SD	39.1 ± 16.5		43.7 ± 15.4		41.3 ± 16.1	
Male	126	43.3	103	38.9	229	41.2
Female	165	56.7	162	61.1	327	58.8
Anterior scleritis	179	61.5	197	74.3	376	67.6
Posterior scleritis	92	31.6	54	20.4	146	26.3
Generalized scleritis	20	6.9	14	5.3	34	6.1
Systemic associated diseases^a^	32	11.0	24	9.1	59	10.6
SLE	1	0.3	1	0.4	2	0.4
IPT	1	0.3	1	0.4	2	0.4
IBD	1	0.3	1	0.4	2	0.4
Psoriasis	2	0.7	2	0.8	4	0.7
GPA	2	0.7	3	1.1	5	0.9
Gout	1	0.3	5	1.9	6	1.1
RP	4	1.4	2	0.8	6	1.1
AS	8	2.7	4	1.5	12	2.2
RA	13	4.5	7	2.6	20	3.6
Healthy controls 710	469		273		742	
Ethnicity	Han Chinese		Han Chinese		Han Chinese	
Mean age ± SD	39.6 ± 10.2		40.4 ± 11.0		39.9 ± 10.5	
Male	245	52.2	132	48.4	377	50.8
Female	224	47.8	141	51.6	365	49.2

*SD* Standard deviation, *SLE* Systemic lupus erythematosus, *IPT* Inflammatory pseudotumor, *IBD* Inflammatory bowel disease, *GPA* Granulomatosis with polyangiitis, *RP* Relapsing polychondritis, *AS* Ankylosing spondylitis, *RA* Rheumatoid arthritis

^a^One patient from North of the Yangtze River was diagnosed with both AS and Gout. Two patients from South of the Yangtze River were diagnosed with both AS and RP

### Genotyping results of tested SNPs

In total, 28 SNPs of 556 scleritis patients and 742 healthy individuals were genotyped and analyzed. None of SNPs deviated from HWE in the healthy volunteers, and the call rate of all SNPs tested was above 90%. Two SNPs, rs9494885 and rs2230926 in TNFAIP3, were correlated with scleritis. Lower frequencies of the T allele and TT genotype of rs9494885 (*P* = 0.047, OR = 0.727, 95% CI = 0.530–0.997; *P* = 0.042, OR = 0.710, 95% CI = 0.510–0.989, respectively) and higher frequencies of the C allele and TC genotype (*P* = 0.047, OR = 1.376, 95% CI = 1.003–1.886; *P* = 0.045, OR = 1.406, 95% CI = 1.007–1.965, respectively) were observed in scleritis patients as compared to the controls (Table 2). Regarding rs2230926, we found that the frequencies of the TT genotype and T allele were decreased in scleritis patients (*P* = 0.037, OR = 0.660, 95% CI = 0.446–0.997; *P* = 0.040, OR = 0.675, 95% CI = 0.464–0.984, respectively), while the G allele and GT genotype frequencies were increased (*P* = 0.040, OR = 1.481, 95% CI = 1.016–2.157; *P* = 0.039, OR = 1.519 95% CI = 1.020–2.263, respectively; Table 2). However, the statistical significance of the weak associations with scleritis was lost after Bonferroni correction (Table 2). As for the remaining 26 SNPs, we did not observe any association with scleritis (Supplementary Table S2).

**Table 2:** Association of two SNPs in TNFAIP3 with scleritis diseases

Genotype/allele	Cases		Controls		*P* value	Pc value	OR (95% CI)
	*n*	%	*n*	%			
rs9494885	*N* = 555		*N* = 739				
TT	474	0.854	659	0.892	0.042	NS	0.710 (0.510–0.989)
TC	79	0.142	78	0.105	0.045	NS	1.406 (1.007–1.965)
CC	2	0.004	2	0.003	0.773	NS	1.333 (0.187–9.491)
T	1027	0.925	1396	0.945	0.047	NS	0.727 (0.530–0.997)
C	83	0.075	82	0.055	0.047	NS	1.376 (1.003–1.886)
rs2230926	*N* = 553		*N* = 724				
GG	2	0.004	2	0.003	0.787	NS	1.310 (0.184–9.331)
GT	56	0.101	50	0.069	0.039	NS	1.519 (1.020–2.263)
TT	495	0.895	672	0.928	0.037	NS	0.660 (0.446–0.977)
G	60	0.054	54	0.037	0.040	NS	1.481 (1.016–2.157)
T	1046	0.946	1394	0.963	0.040	NS	0.675 (0.464–0.984)

*Pc value P* value with Bonferroni correction, *CI* confidence interval, *OR* odds ratio, *TNFAIP3* tumor necrosis factor alpha-induced protein 3. Pc value < 0.05 was regarded to have statistical significance

### Age effects

Due to the significant difference between the average age of the scleritis patients and normal controls, and to exclude the influence of age on the results, we divided the patients and healthy controls into young and old age groups, based on the average age, and then performed statistical analysis to determine whether age affects genotype and allele frequency. The results show that there were no significant differences in genotype and allele frequencies between younger and older people either in patients or healthy controls (Table 3).

**Table 3:** Genotype and allele frequencies of younger and older patients, and younger and older healthy controls

SNP	Genotype/allele	Younger cases		Older cases		*P* value	Pc value	OR (95% CI)
		*n*	Frequency	*n*	Frequency			
rs9494885	TT	213	0.845	261	0.861	0.592	NS	0.879 (0.548–1.409)
	TC	37	0.147	42	0.139	0.783	NS	1.069 (0.663–1.724)
	CC	2	0.008	0	0	0.120	NS	–
	T	463	0.919	564	0.931	0.048	NS	0.841 (0.538–1.316)
	C	41	0.081	42	0.069	0.048	NS	1.189 (0.760–1.860)
rs2230926	GG	1	0.004	1	0.003	0.900	NS	1.195 (0.074–19.206)
	GT	27	0.107	29	0.097	0.675	NS	1.126 (0.647–1.957)
	TT	224	0.889	271	0.900	0.662	NS	0.886 (0.514–1.572)
	G	29	0.058	31	0.051	0.658	NS	1.125 (0.668–1.893)
	T	475	0.942	571	0.949	0.658	NS	0.889 (0.528–1.497)
		Younger controls		Older controls		*P* value	Pc value	OR (95% CI)
		*n*	Frequency	*n*	Frequency			
rs9494885	TT	324	0.885	335	0.898	0.573	NS	0.875 (0.550–1.393)
	TC	42	0.115	36	0.097	0.420	NS	1.213 (0.758–1.943)
	CC	0	0	2	0.005	0.161	NS	–
	T	690	0.943	706	0.946	0.752	NS	0.931 (0.596–1.453)
	C	42	0.057	40	0.054	0.752	NS	1.074 (0.688–1.677)
rs2230926	GG	0	0	2	0.005	0.160	NS	–
	GT	29	0.081	21	0.058	0.217	NS	1.440 (0.805–2.575)
	TT	330	0.919	342	0.937	0.355	NS	0.765 (0.434–1.350)
	G	29	0.040	25	0.034	0.537	NS	1.187 (0.688–2.047)
	T	689	0.960	705	0.966	0.537	NS	0.843 (0.488–1.453)

*Pc value P* value with Bonferroni correction, *CI* Confidence interval, *OR* Odds ratio. Pc value < 0.05 was regarded to have statistical significance

### Stratified analysis

To further confirm whether anatomical location and associated systemic diseases could affect the frequencies of genotypes and alleles in scleritis, we performed a stratified study of the two SNPs described above in various scleritis subgroups compared to normal controls, including patients with anterior, posterior, and pan-scleritis and in patients with or without related systemic diseases. No statistical differences were found in the subgroups of scleritis with the two SNPs after Bonferroni correction (Supplementary Table S3).

### Haplotype analysis

The haplotypes of TNFAIP3 (tumor necrosis factor alpha-induced protein 3), TNFRSF1A (tumor necrosis factor receptor superfamily member 1A), TNFSF4 (tumor necrosis factor ligand superfamily member 4), and TNFSF15 (tumor necrosis factor ligand superfamily member 15) having more than one SNP were analyzed on the SHEsis online platform in our scleritis patients [31]. Haplotypes were not included in the analysis if the frequency was below 3%. A TNFAIP3 TGT haplotype (order of SNPs: rs9494885, rs3799491, rs2230926), a TNFSF4 GT haplotype (order of SNPs: rs3850641, rs704840), and a TNFSF15 CCC haplotype (order of SNPs: rs6478106, rs3810936, rs7865494) showed significant associations with scleritis (Pc = 0.021, OR = 0.717, 95% CI = 0.563–0.913; Pc = 0.004, OR = 1.691, 95% CI = 1.205–2.372; Pc = 0.012, OR = 1.662, 95% CI = 1.168–2.363, respectively) (Tables 4, 5, and 6). On the basis of anatomical location subgroups, stratified analysis showed that the TNFSF4 GC haplotype frequency was increased in the anterior and generalized (pan) scleritis group in comparison with the healthy volunteers group (Pc = 0.022, OR = 1.636, 95% CI = 1.117–2.396; Pc = 4.38 × 10^−4^, OR = 3.744, 95% CI = 1.772–7.910, respectively) (Table 5). The TNFSF15 CCC haplotype frequency was significantly increased in the anterior scleritis group (Pc = 0.003, OR = 1.860, 95% CI = 1.267–2.730), but this was not seen in the other scleritis groups (Table 6). Dividing patients into subgroups with or without systemic diseases, decreased frequency of TNFAIP3 TGT haplotype was observed, whereas elevated frequency of TNFSF4 GT haplotype was found in patients without systemic diseases (Pc = 0.024, OR = 0.715, 95% CI = 0.558–0.916; Pc = 0.004, OR = 1.719, 95% CI = 1.216–2.430, respectively) (Tables 4 and 5). The TNFSF15 CCC haplotype frequency was discovered to be significantly increased in scleritis cases without as well as with systemic diseases, as compared with healthy volunteers (Pc = 0.039, OR = 1.579, 95% CI = 1.096–2.274; Pc = 0.027, OR = 2.496, 95% CI = 1.229–5.067, respectively) (Table 6). The TNFRSF1A haplotype was not significantly correlated with scleritis or its subgroups (Supplementary Table S4).

**Table 4:** Haplotype analysis for association of TNFAIP3 gene polymorphisms with scleritis

	Haplotype^a^	Cases (%)	Controls (%)	*χ*^2^	*P* value	Pc value	OR (95% CI)
Scleritis	CGG	57.95 (0.052)	52.81 (0.037)	3.922	0.048	NS	1.467 (1.002~2.149)
	TAT	93.33 (0.085)	95.70 (0.066)	3.168	0.075	NS	1.390 (0.972~1.761)
	TGT	926.62 (0.839)	1268.28 (0.878)	7.307	0.007	*0.021*	0.717 (0.563~0.913)
Scleritis subgroups							
Anterior scleritis	TGT	593.56 (0.841)	1268.28 (0.878)	5.103	0.024	NS	0.729 (0.554~0.960)
Posterior scleritis	TGT	229.34 (0.837)	1268.28 (0.878)	3.531	0.060	NS	0.695 (0.475~1.017)
Pan-scleritis	TGT	53.00 (0.828)	1268.28 (0.878)	1.518	0.218	NS	0.659 (0.338~1.285)
Scleritis without systemic disease	TGT	835.12 (0.840)	1268.28 (0.878)	7.072	0.008	*0.024*	0.715 (0.558~0.916)
Scleritis with systemic disease	TGT	92.00 (0.836)	1268.28 (0.878)	1.777	0.183	NS	0.699 (0.412~1.187)

Order of SNPs for haplotype analysis is rs9494885, rs3799491, and rs2230926. *TNFAIP3* tumor necrosis factor alpha-induced protein 3, *Pc value P* value with Bonferroni correction, *NS* not significant. Italicized values are statistically significant

^a^Haplotypes with a frequency less than 3% were not included in the analysis

**Table 5:** Haplotype analysis for association of TNFSF4 gene polymorphisms with scleritis

	Haplotype^a^	Cases (%)	Controls (%)	*χ*^2^	*P* value	Pc value	OR (95% CI)
Scleritis	AG	316.16 (0.312)	429.09 (0.315)	0.019	0.891	NS	0.988 (0.829~1.177)
	AT	527.84 (0.522)	730.91 (0.537)	0.529	0.467	NS	0.941 (0.800~1.108)
	GG	88.84 (0.088)	136.91 (0.101)	1.094	0.296	NS	0.861 (0.651~1.140)
	GT	79.16 (0.078)	65.09 (0.048)	9.419	0.002	*0.004*	1.691 (1.205~2.372)
Scleritis subgroups							
Anterior scleritis	GT	49.77 (0.076)	65.09 (0.048)	6.505	0.011	*0.022*	1.636 (1.117~2.396)
Posterior scleritis	GT	16.06 (0.065)	65.09 (0.048)	1.259	0.262	NS	1.379 (0.785~2.424)
Pan-scleritis	GT	9.17 (0.158)	65.09 (0.048)	13.678	2.19 × 10^−4^	*4.38 × 10*^−*4*^	3.744 (1.772~7.910)
Scleritis without systemic disease	GT	72.27 (0.079)	65.09 (0.048)	9.607	0.002	*0.004*	1.719 (1.216~2.430)
Scleritis with systemic disease	GT	6.70 (0.066)	65.09 (0.048)	0.655	0.418	NS	1.402 (0.616~3.188)

Order of SNPs for haplotype analysis is rs3850641 and rs704840. *TNFSF4* tumor necrosis factor ligand superfamily member 4, *Pc value P* value with Bonferroni correction, *NS* not significant. Italicized values are statistically significant

^a^Haplotypes with a frequency less than 3% were not included in the analysis

**Table 6:** Haplotype analysis for association of TNFSF15 gene polymorphisms with scleritis

	Haplotype^a^	Cases (%)	Controls (%)	*χ*^2^	*P* value	Pc value	OR (95% CI)
Scleritis	CCC	71.22 (0.080)	61.93 (0.051)	8.122	0.004	*0.012*	1.662 (1.168~2.363)
	CCT	126.33 (0.143)	187.89 (0.154)	0.342	0.559	NS	0.930 (0.728~1.187)
	CTC	417.41 (0.471)	573.14 (0.470)	0.148	0.701	NS	1.035 (0.869~1.232)
	CTT	37.05 (0.042)	58.03 (0.048)	0.308	0.579	NS	0.888 (0.582~1.353)
	TCC	217.94 (0.246)	335.10 (0.275)	1.63	0.202	NS	0.879 (0.721~1.072)
Scleritis subgroups							
Anterior scleritis	CCC	51.62 (0.089)	61.93 (0.051)	10.319	0.001	*0.003*	1.860 (1.267~2.730)
Posterior scleritis	CCC	13.82 (0.065)	61.93 (0.051)	0.87	0.351	NS	1.331 (0.729~2.432)
Pan-scleritis	CCC	–	–	–	–	–	–
Scleritis without systemic disease	CCC	61.54 (0.077)	61.93 (0.051)	6.113	0.013	*0.039*	1.579 (1.096~2.274)
Scleritis with systemic disease	CCC	9.98 (0.116)	61.93 (0.051)	6.832	0.009	*0.027*	2.496 (1.229~5.067)

Order of SNPs for haplotype analysis is rs6478106, rs3810936, and rs7865494. *TNFSF15* tumor necrosis factor ligand superfamily member 15, *Pc value P* value with Bonferroni correction, *NS* not significant. Italicized values are statistically significant

^a^Haplotypes with a frequency less than 3% were not included in the analysis

## Discussion

In this study, haplotype analysis showed that a TNFAIP3 TGT haplotype, a TNFSF4 GT haplotype, and a TNFSF15 CCC haplotype were significantly associated with scleritis. Any individual SNPs did not show a statistically significant association with scleritis.

Only few studies have analyzed genetic associations with uveitis. A significantly increased frequency of the GG genotype and G allele of rs3087243 in CTLA4 has been reported in scleritis [6]. Haplotype analysis showed a significantly increased prevalence of the TTATACGCG haplotype in PTPN22 and a decreased prevalence of the TCAA haplotype in CTLA4 (order of SNPs: rs3789604, rs150426536, rs1746853, rs12174003, rs1217406, rs3789609, rs1217414, rs3789612, rs2488457; order of SNPs: rs733618, rs5742909, rs231775, rs3087243, respectively) [6].

We made several efforts to validate the experimental data and guarantee the correctness of our experimental results [32]. Firstly, our scleritis patients were selected on the basis of well-defined criteria including the engorgement of the vascular plexus in the superficial and deep episclera and swelling and edema in the scleral tissues. Second, we excluded scleritis patients with a definite infectious etiology. Third, to avoid ethnic bias, only Chinese Han patients and controls were included. Furthermore, to avoid confounding by genetic ancestry, the healthy volunteers were matched according to the geographical regions of the patients.

TNFAIP3 encodes a ubiquitin-editing enzyme A20, working downstream of TNF-α [24]. It inhibits the NF-kB signaling pathway and thus prevents overstimulation of the immune response [24, 33]. In our study, weak associations were detected between two SNPs in the TNFAIP3 gene and scleritis. We found an increased prevalence of the TC genotype and a decreased frequency of the TT genotype in rs9494885 among scleritis patients. This trend has also been reported in Behcet’s disease (BD) [34], allergic rhinitis (AR) [35], and Vogt-Koyanagi-Harada syndrome (VKH) [36] in Chinese Han populations. This variant does not have a direct biological function, since the TNFAIP3 expression was not obviously different in TC genotype as compared to TT genotype carriers of rs9494885 in BD [34]. Moreover, we observed that the TT genotype frequency was lower, while the GT genotype frequency was higher in rs2230926, which is a nonsynonymous variant and leads to a change of the A20 protein at residue 127 from phenylalanine to cysteine [37]. Previous studies also reported similar observations concerning the risk association of scleritis with the G allele and GT genotype, such as RA [38], SLE [28, 39], and primary immune thrombocytopenia (IPT) [40]. Furthermore, the expression of A20 mRNA was higher in rs2230926 GT genotype as compared to TT genotype carriers [41]. GT genotype carriers were more likely to have a poor treatment outcome in RA patients [41]. On the basis of the research findings referred above, we hypothesized that the genotypes that showed an association with other autoimmune disorders might also play a role in the development of scleritis.

Haplotype analysis allows a joint estimation of the role of multiple SNPs in a gene, and it may be more effective than the one-SNP-at-a-time approach to tag uncommon associated variants in catching genetic associations by reducing dimensionality [42]. We observed that one TNFAIP3 haplotype TGT was protective for developing scleritis, and this specific association has to our knowledge not been found in other diseases. The response to TNF blockers has been reported to be significantly associated with the TNFAIP3 TG haplotype (order of SNPs: rs2230926, rs610604) in psoriasis [43]. Additional studies are necessary to investigate whether TNFAIP3 haplotypes might also have an impact on the therapeutic response of scleritis patients to biological agents.

We also found a higher frequency of the TNFSF4 GT haplotype in scleritis patients. The tumor necrosis factor ligand superfamily member 4 (TNFSF4, OX40L) encodes a type II transmembrane protein which can interact with TNFRSF4 (OX40) leading to a costimulatory signal for various immune responses, and which has been put forward as a potential therapeutic target to control autoimmunity [44]. In earlier reports, the GT haplotype of the TNFSF4 gene (order of SNPs: rs844648, rs704840) played a protective role in neuromyelitis optica spectrum disorders in Chinese [45]. The AA haplotype of TNFSF4 (order of SNPs: rs844648, rs10912580) has been shown to be correlated with the occurrence of breast cancer [46]. The biological function of the GT haplotype described in our study has not yet been reported, and its exact role in the pathogenesis of scleritis therefore remains unclear.

Our results showed that a TNFSF15 CCC haplotype was associated with an increased susceptibility to develop scleritis. TNFSF15 encodes the tumor necrosis factor-like ligand 1a (TL1A), which acts as a costimulatory molecule regulating T cell-mediated immunity [47]. The local and systemic levels of TLA1 were significantly increased in scleritis related systemic diseases, such as RA [48], psoriasis [49], IBD [50], AS [51], and SLE [52]. TL1A serum levels were significantly decreased in patients with RA [48] after treatment with an anti-TNF agent and AS patients [51]. Similar results of TNFSF15 haplotype analysis were discovered in IBD [20], breast cancer [53], SLE [54], CD [21], and UC [55], although the composition of the haplotypes was different from that found in our study. How these haplotypes affect the function of TNF related pathways is unclear and deserves further investigation.

## Conclusions

In conclusion, we identified the association of various TNF gene related haplotypes with scleritis in Chinese Han, including TGT in TNFAIP3, GT in TNFSF4, and CCC in TNFSF15.

## Materials and methods

### Subjects

For this project, we recruited 556 patients with scleritis and 742 matched healthy controls at the Department of Ophthalmology of the First Affiliated Hospital of Zhengzhou University (Zhengzhou, China, from May 2017 to November 2019) and the Department of Ophthalmology of the First Affiliated Hospital of Chongqing Medical University (Chongqing, China, from May 2009 to November 2019). The patients with the presence of swelling and edema in the scleral tissues and engorgement of the superficial and deep vascular plexus of the episclera were diagnosed as scleritis, as shown in an earlier paper [1, 2]. Anterior, posterior, and generalized (pan) scleritis were defined following published classification schemes [1, 2]. Fourteen cases were excluded because they were caused by an infection (13 tuberculosis, 1 leprosy). Patients with an unclear diagnosis due to lack of sufficient clinical details, such as a satisfactory B-scan or UBM data, were excluded. All participants were Han Chinese. The 742 enrolled healthy controls were matched with regard to ethnicity, sex, and age.

### SNP selection

A thorough search was performed through a combination of different search terms in the Medline, PubMed, and Ensembl databases, which includes polymorphisms, single-nucleotide polymorphisms (SNPs), genetic polymorphisms, gene polymorphism, TNFAIP3, TNFRSF1A, TNFSF4, TNFSF15, and all the aliases for these four genes. In the end, an aggregate of 405 articles and 49 SNPs were screened (Supplementary Table S5), which were revealed to have a significant association with one or more inflammatory and autoimmune disorders. Using the Han Chinese HapMap database, we checked the linkage disequilibrium (LD) between certain SNPs (*r*^2^ threshold of 0.8) and a known polymorphism in Chinese populations, which resulted in the exclusion of 21 SNP sites, despite the fact that these SNPs have been reported to be associated with the susceptibility of several immune-mediated disorders in other ethnic groups. Seven SNPs lacked a polymorphism in the Chinese population, and 14 SNPs showed a linkage disequilibrium with other SNPs (Supplementary Table S6). After using these criteria, we ended up with a total of 28 SNPs, which included TNFAIP3 (rs7753873, rs5029928, rs9494885, rs610604, rs2230926, rs3799491), TNFRSF1A (rs4149577, rs1800693, rs767455, rs2234649, rs1800692, rs4149570, rs4149578, rs12426675), TNFSF4 (rs12039904, rs1234315, rs2205960, rs704840, rs1234314, rs3850641, rs3861950), and TNFSF15 (rs6478106, rs4979462, rs3810936, rs6478108, rs11554257, rs7865494, rs10817669).

### DNA extraction and genotyping

The QIAamp DNA Blood Mini Kit (Qiagen, Valencia, CA, USA) was used to obtain genomic DNA from peripheral blood collected in EDTA tubes and stored at − 80 °C until used for genotype analysis. The primer sequences of iPLEX reactions were designed through Mass ARRAY® Assay design software (Supplementary Table S1). This experiment was performed strictly according to the guidelines of the manufacturer (Agena Bioscience, San Diego, CA, USA). The iPLEX Gold Assay and TYPER version 4.0.20 software was used to identify and analyze genotypes of patients and controls.

### Statistical analysis

The significant deviation from the Hardy-Weinberg equilibrium (HWE) was calculated using chi-square (*χ*^2^) test in the healthy group. All experimental data concerning differences of candidate SNPs with respect to genotype and allele were evaluated using the *χ*^2^ or Fisher’s exact test by SPSS version 19.0 (SPSS, Chicago, IL, USA). Bonferroni correction was applied to account for multiple testing. This method multiplies the *P* value of the chi-square or Fisher’s exact test by the number of simultaneous tests (*n* = 28) in our study. A Pc (*P* value correction) less than 0.05 was taken as statistically significant.

## Supplementary Information

**Additional file 1: Tables S1–S4.**

**Additional file 2: Table S5.**

**Additional file 3: Table S6.**

## Data Availability

All data generated or analyzed during this study are included in this published article and its supplementary information files.

## Abbreviations

GWAS: Genome-wide association studies
SNP: Single-nucleotide polymorphism
TNF: Tumor necrosis factor
TNFAIP3: Tumor necrosis factor alpha-induced protein 3
TNFSF4: Tumor necrosis factor ligand superfamily member 4
TNFSF15: Tumor necrosis factor ligand superfamily member 15
TNFRSF1A: Tumor necrosis factor receptor superfamily member 1A
AS: Ankylosing spondylitis
RA: Rheumatoid arthritis
MS: Multiple sclerosis
IBD: Inflammatory bowel disease
SSc: Systemic sclerosis
SLE: Systemic lupus erythematosus
BD: Behcet’s disease
AR: Allergic rhinitis
VKH: Vogt-Koyanagi-Harada syndrome
IPT: Primary immune thrombocytopenia
CD: Crohn’s disease
UC: Ulcerative colitis
HWE: Hardy-Weinberg equilibrium
