# Supplementary material for: The haplotypes of various TNF related genes associated with scleritis in Chinese Han
Source: Hum Genomics. 2020 Dec 7;14:46. doi: 10.1186/s40246-020-00296-y (PMC7720609; doi:10.1186/s40246-020-00296-y)
Supplement: Supplementary file 1 — Additional file 1: Tables S1–S4. [file 40246_2020_296_MOESM1_ESM.docx]

CI= confidence interval; OR= odds ratio

*P*c-value= P value with Bonferroni correction

Italicized values are statistically significant

*P*c-value < 0.05 indicates statistical significance

CI= confidence interval; OR= odds ratio

*P*c-value= P value with Bonferroni correction

Italicized values are statistically significant

*P*c-value < 0.05 indicates statistical significance

CI= confidence interval; OR= odds ratio

*P*c-value= P value with Bonferroni correction

Italicized values are statistically significant

*P*c-value < 0.05 indicates statistical significance
